# Screening and association testing of common coding variation in steroid hormone receptor co-activator and co-repressor genes in relation to breast cancer risk: the Multiethnic Cohort

**DOI:** 10.1186/1471-2407-9-43

**Published:** 2009-01-30

**Authors:** Christopher A Haiman, Rachel R Garcia, Chris Hsu, Lucy Xia, Helen Ha, Xin Sheng, Loic Le Marchand, Laurence N Kolonel, Brian E Henderson, Michael R Stallcup, Geoffrey L Greene, Michael F Press

**Affiliations:** 1 Department of Preventive Medicine, Keck School of Medicine, University of Southern California/Norris Comprehensive Cancer Center, Los Angeles, California, USA; 2 Department of Pharmacology and Pharmaceutical Sciences, School of Pharmacy, University of Southern California, Los Angeles, California, USA; 3 Epidemiology Program, Cancer Research Center, University of Hawaii, Honolulu, Hawaii, USA; 4 Department of Biochemistry and Molecular Biology, Keck School of Medicine, University of Southern California/Norris Comprehensive Cancer Center, Los Angeles, California, USA; 5 The Ben May Department for Cancer Research, The University of Chicago, Chicago Illinois, USA; 6 Department of Pathology, Keck School of Medicine, University of Southern California/Norris Comprehensive Cancer Center, Los Angeles, California, USA

## Abstract

**Background:**

Only a limited number of studies have performed comprehensive investigations of coding variation in relation to breast cancer risk. Given the established role of estrogens in breast cancer, we hypothesized that coding variation in steroid receptor coactivator and corepressor genes may alter inter-individual response to estrogen and serve as markers of breast cancer risk.

**Methods:**

We sequenced the coding exons of 17 genes (*EP300, CCND1, NME1, NCOA1, NCOA2, NCOA3, SMARCA4, SMARCA2, CARM1, FOXA1, MPG, NCOR1, NCOR2, CALCOCO1, PRMT1, PPARBP *and *CREBBP*) suggested to influence transcriptional activation by steroid hormone receptors in a multiethnic panel of women with advanced breast cancer (n = 95): African Americans, Latinos, Japanese, Native Hawaiians and European Americans. Association testing of validated coding variants was conducted in a breast cancer case-control study (1,612 invasive cases and 1,961 controls) nested in the Multiethnic Cohort. We used logistic regression to estimate odds ratios for allelic effects in ethnic-pooled analyses as well as in subgroups defined by disease stage and steroid hormone receptor status. We also investigated effect modification by established breast cancer risk factors that are associated with steroid hormone exposure.

**Results:**

We identified 45 coding variants with frequencies ≥ 1% in any one ethnic group (43 non-synonymous variants). We observed nominally significant positive associations with two coding variants in ethnic-pooled analyses (*NCOR2*: His52Arg, OR = 1.79; 95% CI, 1.05–3.05; *CALCOCO1*: Arg12His, OR = 2.29; 95% CI, 1.00–5.26). A small number of variants were associated with risk in disease subgroup analyses and we observed no strong evidence of effect modification by breast cancer risk factors. Based on the large number of statistical tests conducted in this study, the nominally significant associations that we observed may be due to chance, and will need to be confirmed in other studies.

**Conclusion:**

Our findings suggest that common coding variation in these candidate genes do not make a substantial contribution to breast cancer risk in the general population. Cataloging and testing of coding variants in coactivator and corepressor genes should continue and may serve as a valuable resource for investigations of other hormone-related phenotypes, such as inter-individual response to hormonal therapies used for cancer treatment and prevention.

## Background

Breast cancer risk is related to lifetime exposure to steroid hormones.[1-3] Continuous exposure to endogenous and exogenous estrogens enhances cell proliferation in breast tissue, which is thought to increase the chance that a spontaneous mutation may become fixed and lead to a malignant phenotype.[4,5] Inherited polymorphisms in genes involved in steroid hormone biosynthesis may serve as markers of lifetime exposure to elevated levels of estrogen and breast cancer risk.[4,6,7] While studies have demonstrated genetic control of steroid hormone production, associations between common genetic variation and circulating hormone levels have been modest, and insufficient to alter one's risk of developing breast cancer.[8,9]

Cellular response to estrogens is mediated through estrogen receptors (ERα and ERβ), which upon binding to ligand and DNA hormone response elements, recruit coactivator and corepressor proteins that regulate the expression of steroid hormone target genes.[10,11] More than 200 nuclear receptor coactivators and 40 corepressors have been identified http://www.nursa.org/.[11] The relative recruitment of coactivators versus corepressors for a given ligand (e.g. estradiol vs tamoxifen) is tissue specific and may account for agonist vs. antagonist activity of the same ligand in different tissues.[12,13] Polymorphic variants in these mediators of hormonal responsiveness could affect the functional activity of estrogen receptors following stimulation by endogenous or exogenous (i.e. HRT or SERMs) estrogens and lead to differences in steroid hormone sensitivity which could alter one's risk of developing breast cancer.

In this study, we systematically screened the coding exons of steroid hormone receptor coactivator and corepressor genes in a multiethnic panel of women with breast cancer in an attempt to identify and catalogue potentially functional coding polymorphisms that may serve as genetic markers of breast cancer risk. We targeted 17 genes suggested to influence transcriptional activation by steroid hormone receptors (PGR, ERα, ERβ) through direct binding to these receptors or through interactions with other well characterized co-activator/co-repressor protein complexes (E1A binding protein p300 [EP300], cyclin D1 [CCND1], non-metastatic cells 1 protein [NME1], nuclear receptor coactivator 1 [NCoA1], nuclear receptor coactivator 2 [NCoA2], nuclear receptor coactivator 3 [NCoA3], SWI/SNF related, matrix associated, actin dependent regulator of chromatin, subfamily a, member 4 [SMARCA4], and member 2 [SMARCA2], coactivator-associated arginine methyltransferase 1 [CARM1], forkhead box A1 [FOXA1], N-methylpurine-DNA glycosylase [MPG], nuclear receptor co-repressor 1 [NCOR1], nuclear receptor co-repressor 2 [NCOR2], calcium binding and coiled domain 1 [CalCoCo1], protein arginine methyltransferase 1 [PRMT1], peroxisome proliferator-activated receptor binding protein [PPARBP] and CREB binding protein [CREBBP]). To evaluate the relationship between coding variants in these candidate genes and breast cancer risk, we performed association testing in a large nested case-control study within the Multiethnic Cohort Study.[14]

## Methods

### Study population

The Multiethnic Cohort Study (MEC) is a population-based prospective cohort study that was initiated between 1993 and 1996 and includes subjects from various ethnic groups – African-Americans and Latinos primarily from California (mainly Los Angeles) and Native Hawaiians, Japanese-Americans, and European Americans primarily from Hawaii.[14] State driver's license files were the primary sources used to identify study subjects in Hawaii and California. Additionally, in Hawaii, state voter's registration files were used, and, in California, Health Care Financing Administration (HCFA) files were used to identify additional African American men.

All participants (n = 215,251) returned a 26-page self-administered baseline questionnaire that obtained general demographic, medical and risk factor information such as ethnicity, prior medical conditions, family history of various cancers, dietary exposures, smoking, physical activity, body mass index (BMI), and for women, reproductive history and exogenous hormone use. All participants were 45 to 75 years of age at baseline. In the cohort, incident cancer cases are identified annually through cohort linkage to population-based cancer Surveillance, Epidemiology, and End Results (SEER) registries in Hawaii and Los Angeles County as well as to the California State cancer registry. Information on stage of disease and estrogen and progesterone receptor status is also obtained through the SEER registries.

#### Nested case-control study of breast cancer

Blood sample collection in the MEC began in 1994 and targeted incident breast cancer cases and a random sample of study participants to serve as controls for genetic analyses. In this present study, incident cases are defined as those diagnosed with invasive breast cancer after enrollment through December 31, 2002. Cases were over 45 years of age, and consisted primarily of postmenopausal women. Women with a previous diagnosis of breast cancer identified by SEER or self-report at baseline were excluded. Controls were women without a breast cancer diagnosis through December 31, 2002. The controls were frequency matched to cases on ethnicity and the case's age at diagnosis in 5-year intervals. The nested breast cancer case-control study consists of 1,612 invasive breast cancer cases and 1,961 controls (n, cases/n, controls: African Americans, 345/426; Native Hawaiians, 108/290; Japanese Americans, 425/419; Latinas 334/386; European Americans, 400/440), and has been utilized previously for numerous candidate gene association studies in the MEC. [15-17] This study was approved by the Institutional Review Boards at the University of Southern California and at the University of Hawaii and informed consent was obtained from all study participants

### Laboratory methods

#### Polymorphism discovery

Polymorphism discovery was carried out by sequencing the coding exons and splice-site regions of the 17 candidate genes in a multiethnic panel of 95 women (19 subjects of each ethnic group) with advanced breast cancer (invasive/non-localized cancer with SEER stage ≥ 2) from the MEC. These genes were selected because they are the focus of ongoing structural and functional studies being conducted by the investigators. Advanced cases were targeted for sequencing to increase the likelihood of detecting variants that would be biologically associated with breast cancer. This panel was selected to have ≥ 85% power to detect a potentially functional variant of ~5% frequency (2 of 38 chromosomes) in any one population or an overall frequency of ~1% (2 of 190 chromosomes).

DNA was extracted from buffy coat fractions using the Qiagen QiaAMP Blood Kit (Valencia, CA). All DNA samples were previously whole-genome amplified (WGA) by Molecular Staging Inc. following their standard protocol (New Haven, CT).[18]

Non-synonymous variants in the coding region or variants in known splice-site regions that were observed in > 1 individual (a minimum of 2 out of the 190 chromosomes) were targeted for association testing. For variants that were observed in only one individual, we sequenced an additional 88–91 subjects of that specific ethnic population. This extra sequencing was performed to determine whether the variant is extremely rare or may have been introduced during the WGA process or through PCR-based sequencing, with each having error rates of ~10^-6^.[19] Of the 68 rare ethnic-specific variants that we observed, 18 were confirmed (i.e. observed in ≥ 1 of the additional 190 chromosomes and ≥ 2 of 228 chromosomes examined in total (~1%)), and were further examined in relation to breast cancer risk.

#### DNA sequencing

Bi-directional sequencing was performed on the ABI 3730 × l DNA Analyzer (Applied Biosystems, Foster City, CA). Gene and exon specific PCR primers were obtained from NCBI Probe Database http://www.ncbi.nlm.nih.gov/sites/entrez?cmd=search&db=probe when available, and PCR conditions are according to the VariantSEQr Resequencing System protocol (Applied Biosystems, Foster City, CA). In the event that PCR primers were not available (13 exons out of 353 targeted exons), primers were designed in-house (at least 50 bases upstream and downstream from the targeted exon). In the instance that an exon exceeded approximately 550 bases or sequencing did not yield analyzable results, internal primers were designed. Sequencing primers were typically universal primers obtained from ABI or internal primers, as mentioned above. Sequencing purification was performed using DyeDX 96 columns (Qiagen, Valencia, CA) following their standard protocol. PCR primers, cycling conditions and details of the sequencing protocol can be provided upon request.

PolyPhred was used for analyzing sequence traces and variation discovery http://droog.mbt.washington.edu/PolyPhred.html. [20-22] For the 17 genes evaluated in this study (Table 1), we successfully sequenced 346 of the 355 coding exons (97.5%; > 72 kilobases (kb)), 327 in both the forward and reverse direction, and 19 in only one direction (a total of 367 amplicons). Each amplicon was sequenced in 94 of the 95 subjects in the multiethnic panel, on average. The Phred quality score was used to assess the quality of each trace from 10 bases 5' through 10 bases 3' of each exon.[23] The average Phred quality score was 46.6 for all exons sequenced; 86% of the amplicons had a quality score ≥ 40 and 97.5% had a quality score ≥ 30.

**Table 1:** Candidate Estrogen Receptor Coactivator and Corepressor-related Genes.

Gene Symbol	Gene Name	Chromosome	NM Number^a^	Total Exons (Coding)
**EP300**	E1A Binding Protein p300	22q13	NM_001429	31 (31)
**CCND1**	cyclin D1	11q13	NM_053056	5 (5)
**NME1**	non-metastatic cells 1, protein (NM23A)	17q21	NM_000269	5 (4)
**NCOA1**	nuclear receptor coactivator 1	2p23	NM_003743	21 (19)
**NCOA2**	nuclear receptor coactivator 2	8q13	NM_006540	23 (21)
**NCOA3**	nuclear receptor coactivator 3	20q13	NM_181659	23 (21)
**SMARCA4**	SWI/SNF related, matrix associated, actin dependent regulator of chromatin, subfamily a, member 4	19p13	NM_003072	35 (34)
**SMARCA2**	SWI/SNF related, matrix associated, actin dependent regulator of chromatin, subfamily a, member 2	9p24	NM_003070	34 (33)
**CARM1**	coactivator-associated arginine methyltransferase 1	19p13	NM_199141	16 (16)
**FOXA1**	forkhead box A1	14q21	NM_004496	2 (2)
**MPG**	N-methylpurine-DNA glycosylase	16p13	NM_002434	5 (4)
**NCOR1**	nuclear receptor co-repressor 1	17p11-p12	NM_006311	46 (45)
**NCOR2**	nuclear receptor co-repressor 2	12q24	NM_006312	48 (47)
**CALCOCO1**	calcium binding and coiled-coil domain 1	12q13	NM_020898	15 (14)
**PRMT1**	protein arginine methyltransferase 1	19q13	NM_001536	11 (11)
**PPARBP**	peroxisome proliferator-activated receptor binding protein	17q12	NM_004774	17 (17)
**CREBBP**	CREB binding protein	16p13	NM_004380	31 (31)

^a ^RefSeq accession number of RNA transcript in NCBI

#### Genotyping

The genotype of coding variants (43 non-synonymous SNPs, 1 in/del and 1 splice-site variant) in the case-control samples was determined using the allelic discrimination assay.[24] Each assay was validated initially by genotyping the multiethnic sequencing panel (n = 95) and comparing with the sequencing results; the concordance was > 99.6%, on average. Five variants could not be genotyped in the case-control samples because a working assay could not be designed (*FOXA1*, Gly227Glu; *NCOR2*, Tyr19Cys, Pro975Ser and Pro2008Ser (rs2230944); *SMARCA2*, Gly1416Ala (rs3793510)). We could infer genotypes at Pro2008Ser in *NCOR2 *however as this variant was found to be in near perfect linkage disequilibrium (i.e. r^2 ^= 1) with Ala2007Thr (determined based on sequencing of 69–84 individuals from each of the 5 populations). The association results for the splice-site variant in *CCND1 *(rs603965, Pro241Pro) in the MEC is also part of another study (Knudsen et al. in review, 2008). Primers and probes for all assays can be provided upon request. Quality control replicates (~5%) were included to assess the genotyping reliability and reproducibility for the 40 assays. The average concordance for the duplicates was 99.8%, ranging from 98%–100% across all assays. All variants were successfully genotyped in > 94% of cases or controls (average call rate = 98.2%)

This study has been approved by the Institutional Review Boards at the University of Southern California and the University of Hawaii.

### Statistical analyses

Hardy-Weinberg equilibrium was evaluated using an exact test.[25] Unconditional logistic regression was used to assess the association of each variant with breast cancer risk. Allele dosage effects were examined using a log-additive model in both ethnic-specific and pooled analyses, treating the common homozygous (i.e. wild-type) genotype class as the "low risk" group. Logistic regression models were fitted to estimate odds ratios associated with this score variable treated as a linear variable, adjusted for age and race. We also examined effect heterogeneity by breast cancer phenotypes (e.g. stage and estrogen receptor (ER) status). These analyses were performed using the standard case-control approach, limiting the cases to those with a specific phenotype (i.e. ER-positive cases) and all controls, and a case-only analysis to test for differences by disease subgroup. We also examined effect modification by established breast cancer risk factors that are associated with steroid hormone exposure: body mass index (kg/m^2^) among postmenopausal women (≥ 25 vs < 25 kg/m^2^), use of hormone replacement therapy (current vs past vs never user of estrogen or estrogen + progestin) and age at menarche (≤ 12, vs > 12 years). Tests for interactions were performed using the Likelihood Ratio Test (LRT). All statistical analyses were done using SAS version 9.0 (Cary, NC).

## Results

### Discovery of coding variation

In resequencing of the 17 candidate genes (Table 1), we identified 43 non-synonymous SNPs with frequencies ≥ 1% in any one ethnic group and 19 of these variants were novel (see Additional file 1, Supplementary table 1). We detected all common (≥ 5%) validated non-synonymous SNPs in these genes reported in dbSNP. We also detected the well known and common splice-site variant in *CCND1 *(rs603965, Pro241Pro).[26] as well as a novel in-frame deletion (Val1996/1997del) in *NCOR1 *in African Americans (MAF, 0.03). Six of the candidate genes targeted in this study were quite large and included > 30 coding exons (Table 1), with four of these genes containing > 7.2 kb of coding sequence (*EP300*, *NCOR1*, *NCOR2 *and *CREBBP*). *NCOR2 *contained both the largest number of coding exons (n = 47, 7.6 kb) as well as non-synonymous variants (n = 15). In contrast, *NCOR1*, an equally large gene was found to harbor no non-synonymous variants (among the 44 of 45 coding exons that were successfully sequenced, 7.3 kb). We also identified three known poly-glutamine repeat polymorphisms, one in exon 20 of *NCOA3*.[27], one in exon 15 of *NCOR2*.[28], and one in exon 4 of *SMARCA2*[29]; associations with these repeat polymorphisms and breast cancer risk will be examined in future studies. All non-synonymous, in-frame in/del and splice-site variants were targeted for association testing (as discussed in the methods) in the breast cancer case-control study in the MEC (1,612 cases and 1,961 controls). A detailed list of the coding variants examined in the breast cancer study, and their frequencies in each racial/ethnic population is provided (see Additional file 1, Supplementary table 1). Only one variant was found in a single population (*NCOA3*, Met391Val; African Americans, MAF 1.6%) and all variants were in Hardy-Weinberg Equilibrium among controls (p > 0.05) in at least four of the five ethnic groups.

#### Allelic associations with breast cancer risk

The median age of the breast cancer cases and controls was 65 and 63 years, respectively. The associations with established breast cancer risk factors for each ethnic group were generally as expected. Briefly, among postmenopausal women, compared to controls, cases were more likely to be heavier and to have used hormone therapy. Cases also reported having an earlier age of menarche and were more likely to report a family history of breast cancer, than controls (Additional file 1, Supplementary Table 2).

The associations of each coding variant with breast cancer risk are presented in Table 2. We observed nominally significant positive associations (p ≤ 0.05) with two variants in ethnic-pooled analyses (*NCOR2*: His52Arg, OR = 1.79; 95% CI, 1.05–3.05; *CALCOCO1*: Arg12His, OR = 2.29; 95% CI, 1.00–5.26). His52Arg in *NCOR2 *was also found to be significantly positively associated regional/metastatic breast cancer (OR = 2.25; 95% CI, 1.15–4.38) while Arg12His in *CALCOCO1 *was more closely associated with ER-positive breast cancer (OR = 2.83; 95% CI, 1.19–6.74) and regional/metastatic disease (OR = 3.19; 95% CI, 1.12–9.11). Associations were not statistically significantly different between disease subgroups (stage or ER status) however for either variant. Both of these variants were relatively rare among controls in each population, with MAFs of ≤ 2.6% (1 homozygous carrier) and ≤ 0.5% (0 homozygous carriers) for His52Arg and Arg12His, respectively. The associations with each coding variant by race-ethnicity and the genotype counts for each variant are provided (see Additional file 1, Supplementary tables 1 and 3).

**Table 2:** The Association of Coding Variants in Candidate Coactivator and Corepressor-related Genes with Breast Cancer Risk.

Gene/Variant	MAF, range^a^	All Groups 1,612 cases/1,961 controls	ER+ 1090 cases	ER- 293 cases	Localized 1187 cases	Regional/Metastatic 420 cases
		**OR(95% CI)^b^**	**OR(95% CI)^b^**	**OR(95% CI)^b^**	**OR(95% CI)^b^**	**OR(95% CI)^b^**
**EP300**						
Ser507Gly	0–0.020	1.01(0.55–1.85)	0.73(0.35–1.54)^c^	2.61(1.15–5.92)^c^	1.01(0.53–1.95)	1.05(0.39–2.83)
Ile997Val	0.023–0.483	0.92(0.81–1.04)	0.86(0.74–0.99)	1.08(0.86–1.36)	0.94(0.82–1.08)	0.87(0.71–1.06)
Pro1986Leu	0–0.007	0.88(0.21–3.73)	1.12(0.26–4.78)	-^d^	0.67(0.13–3.57)	1.35(0.16–11.56)
Gln2223Pro	0–0.036	1.03(0.70–1.52)	1.02(0.66–1.57)	0.91(0.43–1.93)	0.95(0.62–1.47)	1.31(0.75–2.27)
	
**CCND1**						
Pro241Pro	0.244–0.539	1.07(0.97–1.18)	1.12(1.00–1.26)	1.04(0.86–1.26)	1.08(0.96–1.20)	1.06(0.90–1.24)
	
**NCOA1**						
Pro1272Ser	0–0.022	1.11(0.69–1.78)	1.04(0.60–1.79)	1.09(0.46–2.59)	1.31(0.80–2.14)	0.62(0.24–1.58)
	
**NCOA2**						
Ala407Ser	0–0.005	2.25(0.73–6.96)	2.09(0.55–7.89)	2.85(0.67–12.13)	1.23(0.29–5.24)	4.21(1.19–14.85)
Asn1212Ser	0–0.012	1.08(0.45–2.56)	1.27(0.49–3.34)	1.37(0.37–5.00)	0.91(0.33–2.50)	1.56(0.49–4.99)
Met1282Ile	0.019–0.142	1.06(0.87–1.29)	1.07(0.86–1.33)	0.99(0.67–1.46)	1.10(0.89–1.36)	0.94(0.67–1.31)
	
**NCOA3**						
Arg218Cys	0–0.079	0.88(0.68–1.13)	0.90(0.68–1.21)	0.84(0.53–1.33)	0.77(0.58–1.03)	1.13(0.79–1.62)
Met391Val	0–0.016	0.93(0.41–2.13)	1.25(0.49–3.18)	0.33(0.04–2.54)	0.93(0.37–2.35)	0.87(0.24–3.09)
Pro559Ser	0–0.037	1.13(0.68–1.89)	1.48(0.84–2.61)	0.77(0.29–2.03)	1.35(0.78–2.32)	0.65(0.25–1.72)
Gln586His	0.020–0.086	1.00(0.80–1.23)	0.97(0.77–1.24)	1.16(0.80–1.69)	1.07(0.85–1.34)	0.84(0.58–1.20)
Ser662Phe	0–< 0.001	-^d^	-^d^	-^d^	-^d^	-^d^
	
**FOXA1**						
Ala83Thr	0.118–0.580	0.99(0.89–1.10)	0.99(0.88–1.12)	1.07(0.88–1.30)	0.94(0.83–1.06)^c^	1.14(0.96–1.35)^c^
Ser448Asn	0.002–0.057	1.09(0.84–1.42)	1.29(0.97–1.71)	0.69(0.39–1.22)	0.99(0.74–1.33)^c^	1.39(0.96–2.02)^c^
**MPG**						
Val242Leu	0–0.002	1.62(0.36–7.29)	2.70(0.59–12.26)	-^d^	0.61(0.06–5.95)	4.32(0.86–21.62)
	
**NCOR1**						
Val1996/1997del	0–0.028	0.96(0.52–1.77)	0.93(0.44–1.96)	1.22(0.50–2.98)	0.85(0.41–1.74)	1.24(0.53–2.87)
	
**NCOR2**						
Thr35Met	0–0.025	0.18(0.02–1.37)	0.22(0.03–1.72)	-^d^	-^d^	0.71(0.09–5.59)
His52Arg	0–0.026	1.79(1.05–3.05)	1.52(0.81–2.86)	2.09(0.97–4.50)	1.57(0.84–2.96)	2.25(1.15–4.38)
Gly783Glu	0.001–0.125	1.16(0.97–1.39)	1.19(0.98–1.46)	1.03(0.74–1.44)	1.21(0.99–1.46)	1.05(0.79–1.40)
Lys980Thr	0–0.014	1.33(0.67–2.67)	1.16(0.54–2.52)	3.34(1.17–9.53)	1.43(0.69–2.98)	1.16(0.33–4.09)
Ala995Gly	0–0.078	0.95(0.67–1.36)	0.98(0.64–1.50)	1.13(0.66–1.93)	0.99(0.67–1.47)	0.83(0.47–1.46)
Ser1525Thr	0–0.028	0.91(0.51–1.62)	0.75(0.35–1.59)	1.48(0.69–3.20)	0.82(0.42–1.59)	1.16(0.50–2.66)
Ala1706Thr	0.060–0.209	0.99(0.86–1.13)	0.99(0.85–1.15)	0.99(0.77–1.26)	0.96(0.83–1.12)	1.02(0.83–1.26)
Ala2007Thr	0.012–0.049	1.00(0.78–1.29)	0.93(0.69–1.25)	0.97(0.60–1.55)	1.03(0.78–1.36)	0.95(0.63–1.44)
Ala2011Val	0–0.011	1.15(0.48–2.74)	1.65(0.67–4.06)	0.58(0.07–4.60)	0.89(0.30–2.65)	1.83(0.61–5.49)
Thr2216Pro	0–0.022	1.04(0.56–1.93)	1.14(0.57–2.30)	1.02(0.34–3.03)	1.14(0.58–2.22)	0.84(0.28–2.48)
Ser2311Gly	0.001–0.060	0.93(0.64–1.34)	0.90(0.60–1.36)	0.73(0.32–1.67)	1.08(0.73–1.58)^c^	0.48(0.21–1.09)^c^
Ala2496Thr	0–0.062	0.96(0.68–1.38)	1.01(0.69–1.49)	0.57(0.23–1.41)	1.12(0.77–1.63)^c^	0.46(0.20–1.05)^c^
	
**CALCOCO1**						
Arg12His	0–0.005	2.29(1.00–5.26)	2.83(1.19–6.74)	0.76(0.09–6.09)	1.89(0.75–4.75)	3.19(1.12–9.11)
Arg393Lys	0.140–0.365	0.93(0.82–1.05)	0.92(0.80–1.05)	1.01(0.80–1.26)	0.90(0.79–1.03)	0.98(0.80–1.19)
Ala527Thr	0–0.014	0.92(0.41–2.11)	0.82(0.29–2.31)	0.34(0.04–2.62)	1.21(0.51–2.84)	0.29(0.04–2.24)
Gly561Val	0–0.011	1.17(0.57–2.39)	1.34(0.61–2.93)	0.81(0.18–3.60)	1.23(0.56–2.68)	1.10(0.36–3.32)
Thr639Pro	0–0.067	0.95(0.64–1.42)	1.00(0.63–1.60)	0.64(0.30–1.37)	0.84(0.53–1.33)	1.26(0.72–2.20)
	
**CREBBP**						
Pro858Ser	0–0.006	0.67(0.16–2.81)	0.76(0.15–3.98)	1.12(0.13–9.77)	0.63(0.12–3.26)	0.87(0.10–7.57)
Thr910Ala	0–0.002	1.11(0.27–4.48)	1.58(0.39–6.49)	-^d^	1.17(0.26–5.34)	1.11(0.12–10.01)
Val992Ile	0–0.036	1.14(0.68–1.92)	0.60(0.28–1.28)^c^	2.20(1.10–4.39)^c^	1.19(0.67–2.12)	1.10(0.49–2.45)
Gly2229Ser	0–0.011	1.69(0.70–4.07)	1.66(0.61–4.53)	1.09(0.23–5.15)	1.58(0.60–4.17)	1.92(0.58–6.35)
	
**SMARCA2**						
Asp1546Glu	0.129–0.242	1.07(0.94–1.21)	1.12(0.98–1.29)	0.92(0.73–1.16)	1.07(0.93–1.22)	1.07(0.88–1.30)

^a ^Minor allele frequency among controls across populations.

^b ^Adjusted for age and race. OR for gene dosage effects.

^c ^Case-only analysis: ER+ vs ER-, p < 0.01; Localized vs Regional/Metastatic, p < 0.05

^d ^OR can not be calculated because of small numbers of cases or controls.

Allelic associations were also examined by ER status and stage of disease. We observed significant positive associations with single variants in *EP300 *(Ser507Gly), *NCOR2 *(Lys980Thr) and *CREBBP *(Val992Ile) among ER-negative tumors (Table 2), and significant heterogeneity by ER status in case-only analyses (p < 0.01) for Ser507Gly and Val992Ile. There was a statistically significant positive association with *CCND1 *(Pro241Pro) and an inverse association with *EP300 *(Ile997Val) for ER-positive cases (Table 2). In addition to the previously noted associations with His52Arg in *NCOR2 *and Arg12His in *CALCOCO1*, we observed a significant positive association with Ala407Ser in *NCOA2 *and advanced disease and nominally significant heterogeneity (p < 0.05) by disease stage for variants Ala83Thr and Ser448Asn in *FOXA1 *and variants Ser2311Gly and Ala2496Thr in *NCOR2*.

We also evaluated allelic effect modification by known breast cancer risk factors that are associated with steroid hormone exposure, with the *a priori *hypothesis being that conditions related to long-term steroid hormone exposure (i.e. greater postmenopausal weight, early age at menarche and ever use of hormone therapy) may influence the penetrance associated with these candidate coding variants (see Additional file 1, Supplementary tables 4–6). We observed very little evidence to support this hypothesis and only two nominally statistically significant interactions (Ala2007Thr in *NCOR2 *and age at menarche (LRT, p = 0.04); and Ala83Thr in *FOXA1 *and BMI (LRT, p = 0.00054)). Of note we observed a borderline significant positive association with the common Pro241Pro variant in *CCND1 *among current users of HRT (OR = 1.21; 95% CI, 1.00–1.46), however no significant interactions were observed by postmenopausal hormone use status (see Additional file 1, Supplementary table 6).

## Discussion

Inherited susceptibility to breast cancer is associated with a wide spectrum of allelic variants that convey varying degrees of risk (reviewed in [30]). Rare mutations in the coding sequence of *BRCA1 *and *BRCA2 *and a growing number of other genes involved in maintaining genomic stability have been shown to confer high to moderate risks of breast cancer. More recently, genome-wide scans of breast cancer in populations of European ancestry have identified a limited number of common alleles associated with more modest risks of breast cancer (OR ~1.1–1.2 per allele). [31-34] Genome-wide scanning approaches have been shown to be powerful for discovering common variants that influence complex disease phenotypes; however, these approaches currently fail to comprehensively survey coding variation, particularly uncommon alleles in non-European populations. At present, the only way to fully enumerate and comprehensively assess the coding-variant model for breast cancer susceptibility is by direct resequencing of candidate loci. This candidate gene resequencing strategy has been successful in identifying rare truncating mutations in a number of genes that confer approximately 2-fold risks of breast cancer (e.g. *PALB2 *and *BRIP1*).[35,36] This approach has also been successful for identifying rare alleles that act collectively to influence other complex traits and cancer phenotypes, with examples including variants in candidate genes that influence plasma lipid levels [37] and risk of developing colorectal adenomas.[38]

In this study, we examined the role of coding variation in breast cancer in multiple populations, focusing on a set of 17 candidate steroid hormone coactivator and corepressor-related genes selected based on their ability to interact with the estrogen receptor transcription complex and potentially modulate response to estrogen in breast tissue. Sequencing of the coding exons in a multi-ethnic panel of 95 women with advanced breast cancer provided 85% power to identify putative functional coding variants with frequencies as low as 1% in the combined sample. The breast cancer case-control study utilized for association testing was also well-powered to detect nominally significant effects as low as 1.8 for rare alleles (1% MAF, 81% power) and 1.35 for more common alleles (5% MAF, 83% power). This study was also well-powered to detect effects as low as 1.36 for more common alleles (10% MAF, 82% power) after correcting for the number of tests performed (n = 40, α = 0.00125). The multiethnic nature of this study was designed to allow for investigating a wide range of risk alleles; however, we found the vast majority of coding variants to be present in more than one population (39 of 40 variants). In this study, we observed nominally significant associations with only 2 variants and breast cancer risk (His52Arg in *NCOR2 *and Arg12His in *CALCOCO1*), which is in line with expectation based on the number of tests that were performed (2 of 40 = 5%). The observation that these variants were also significantly associated with stage and ER status will need to be confirmed in other studies.

Aside from *CCND1*, coding variation in only a small number of these genes has been investigated in relation to breast cancer risk [39,40]. The Pro241Pro splice-site variant in *CCND1 *has been examined extensively, with some studies reporting a positive association, [41-43] and others finding no significant association [44-46]. We observed no significant association with this variant, and much larger collaborative efforts, such as the Breast Cancer Association Consortium [47], will be needed to rule out weak effects (RR < 1.15) for this functional variant. Our data support those of Wirtenberger et al.[40] who reported no association with the Ile997Val or Gln2223Pro variants in *EP300*. However, we did observe nominally significant associations between Pro241Pro in *CCND1 *and Ile997Val in *EP300 *and risk of ER-positive breast cancer. These findings are noteworthy as both of these variants are relatively common in the population and will need to be confirmed in other large studies. An inverse association has also been reported with the Gln586His variant in *NCOA3 *(p = 0.03) in a European study (775 cases and 1,628 controls), which we were unable to replicate in our much larger study [39]. The glutamine repeat polymorphism in *NCOA3 *has also been investigated in multiple populations, with the vast majority of studies reporting no significant correlation between repeat genotype and breast cancer incidence. [48-52]

Although we conducted a comprehensive assessment of coding variants in these candidate genes, there are a number of limitations to our study that should be considered when interpreting our findings. First, our resequencing strategy most likely missed rare coding variants in these genes, including those which may be population- or subject-specific. Second, we did not enrich our sequencing panel with cases with a family history of breast cancer who may be more genetically susceptible and for whom a coding-variant genetic model may be more probable. Nor did we enrich our panel with cases with ER-positive tumors which may increase the likelihood for detecting putative functional coding variants in these genes based on their known modes of action. Most of the variants that we identified were rare, so power was limited. Future studies of these loci and other steroid hormone receptor coactivator and corepressor genes will require even larger samples from these defined population subgroups to ensure complete ascertainment of all rare coding alleles, followed by robust association studies with greater power to assess more modest effects.

## Conclusion

This study suggests that common coding variation in these 17 candidate steroid hormone receptor coactivator and corepressor genes does not make a substantial contribution to breast cancer risk in the general population. Cataloging and testing of coding variants in coactivator and corepressor genes should continue and may serve as a valuable resource for investigations of other hormone-related phenotypes, such as mammographic density, and of inter-individual response to hormonal therapies used for cancer treatment and prevention.

## Abbreviations

MEC: Multiethnic Cohort; OR: odds ratio; CI: confidence interval; ER: estrogen receptor; BMI: body mass index; WGA: whole-genome amplification; SEER: Surveillance, Epidemiology, and End Results; MAF: minor allele frequency; LRT: likelihood ration test; kb: kilobase

## Competing interests

The authors declare that they have no competing interests.

## Authors' contributions

RG, LX and HH carried out the genotyping and sequencing. LX and XS performed the bioinformatic analyses and data cleaning. RG and CH performed the statistical analyses. LM, LK, BH, MS, GG and MP contributed to the conception and design of the study and provided critical revision of the manuscript for important intellectual content. CAH conceived of the study and coordinated all laboratory work and data analysis. The manuscript was drafted by RG and CH. All authors read and approved of the final manuscript.

## Pre-publication history

The pre-publication history for this paper can be accessed here:

http://www.biomedcentral.com/1471-2407/9/43/prepub

## Supplementary Material

Additional file 1 **Supplementary Tables.** Supplementary tables 1–6. Click here for file
