# Supplementary material for: Screening and association testing of common coding variation in steroid hormone receptor co-activator and co-repressor genes in relation to breast cancer risk: the Multiethnic Cohort
Source: BMC Cancer. 2009 Jan 30;9:43. doi: 10.1186/1471-2407-9-43 (PMC2637888; doi:10.1186/1471-2407-9-43)
Supplement: Additional file 1 — Supplementary Tables. Supplementary tables 1–6. [file 1471-2407-9-43-S1.doc]

**Supplementary Table 1: Association Results, Minor Allele Frequencies and Hardy Weinberg Equilibrium Testing by Ethnic Group.**

|  |  |  | **African Americans** | **Native Hawaiians** | **Japanese Americans** | **Latina** | **European Americans** |  |  |  |
| --- | --- | --- | --- | --- | --- | --- | --- | --- | --- | --- |
| **Genes**  **Sequenced** | **Variant**  **RS#^a^** |  | **cases/controls**  **345/426** | **cases/controls**  **108/290** | **cases/controls**  **425/419** | **cases/controls**  **334/386** | **cases/controls**  **400/440** | **Pooled**  **OR(95%CI)^b^** | **P_Trend_^c^** | **P_Het_^d^** |
| **EP300** | Ser507Gly | HWE | -/- | 0.99/0.99 | 0.15/0.99 | 0.99/0.99 | -/- |  |  |  |
|  |  | MAF | 0/0 | 0.009/0.005 | 0.020/0.020 | 0.002/0.004 | 0/0 |  |  |  |
|  |  | OR(95%CI) | - | 1.65(0.27-10.11) | 1.01(0.52-1.97) | 0.40(0.04-3.87) | - | 1.01(0.55-1.85) | 0.97 | 0.60 |
|  | Ile997Val | HWE | 0.78/0.38 | 0.99/0.82 | 0.99/0.99 | 0.10/0.31 | 0.72/0.83 |  |  |  |
|  | rs20551 | MAF | 0.105/0.092 | 0.103/0.151 | 0.029/0.023 | 0.462/0.483 | 0.291/0.324 |  |  |  |
|  |  | OR(95%CI) | 1.15(0.83-1.61) | 0.64(0.39-1.05) | 1.31(0.71-2.43) | 0.92(0.76-1.13) | 0.85(0.69-1.05) | 0.92(0.81-1.04) | 0.17 | 0.24 |
|  | Pro1986Leu | HWE | -/- | 0.99/0.99 | 0.99/0.99 | -/- | -/- |  |  |  |
|  |  | MAF | 0/0 | 0.009/0.007 | 0.001/0.002 | 0/0 | 0/0 |  |  |  |
|  |  | OR(95%CI) | - | 1.50(0.27-8.41) | 0.45(0.04-5.02) | - | - | 0.88(0.21-3.73) | 0.86 | 0.45 |
|  | Gln2223Pro | HWE | 0.99/0.99 | 0.99/0.99 | 0.99/- | 0.99/0.99 | 0.46/0.43 |  |  |  |
|  | rs1046088 | MAF | 0.010/0.009 | 0.005/0.009 | 0.001/0 | 0.015/0.018 | 0.039/0.036 |  |  |  |
|  |  | OR(95%CI) | 1.13(0.40-3.16) | 0.48(0.05-4.20) | - | 0.84(0.37-1.91) | 1.12(0.67-1.86) | 1.03(0.70-1.52) | 0.86 | 0.94 |
| **CCND1** | Pro241Pro | HWE | 0.11/0.59 | 0.12/0.90 | 0.07/0.62 | 0.49/0.51 | 0.04/0.92 |  |  |  |
|  | **rs603965** | MAF | 0.248/0.244 | 0.525/0.539 | 0.487/0.480 | 0.401/0.384 | 0.516/0.473 |  |  |  |
|  |  | OR(95%CI) | 1.01(0.79-1.27) | 0.96(0.70-1.32) | 1.02(0.84-1.25) | 1.07(0.86-1.33) | 1.24(1.01-1.53) | 1.07(0.97-1.18) | 0.18 | 0.66 |
| **NCOA1** | Pro1272Ser | HWE | 0.99/0.99 | 0.99/0.99 | -/- | 0.99/0.99 | 0.26/0.18 |  |  |  |
|  | rs1804645 | MAF | 0.004/0.005 | 0.009/0.010 | 0/0 | 0.009/0.010 | 0.028/0.022 |  |  |  |
|  |  | OR(95%CI) | 0.95(0.21-4.30) | 0.77(0.15-3.95) | - | 0.88(0.30-2.59) | 1.31(0.71-2.40) | 1.11(0.69-1.78) | 0.66 | 0.93 |
| **NCOA2** | Ala407Ser | HWE | 0.99/0.99 | -/- | -/- | 0.99/0.99 | -/- |  |  |  |
|  |  | MAF | 0.009/0.005 | 0/0 | 0/0 | 0.003/0.001 | 0/0 |  |  |  |
|  |  | OR(95%CI) | 1.98(0.55-7.15) | - | - | 2.47(0.22-27.56) | - | 2.25(0.73-6.96) | 0.16 | 0.90 |
|  | Asn1212Ser | HWE | 0.99/0.99 | -/- | -/- | 0.99/0.99 | -/- |  |  |  |
|  |  | MAF | 0.013/0.012 | 0/0 | 0/0 | 0.001/0.001 | 0/0 |  |  |  |
|  |  | OR(95%CI) | 1.10(0.44-2.74) | - | - | 1.18(0.07-19.02) | - | 1.08(0.45-2.56) | 0.87 | 0.91 |
|  | Met1282Ile | HWE | 0.34/0.99 | 0.99/0.23 | 0.99/0.42 | 0.99/0.99 | 0.69/<0.01 |  |  |  |
|  | rs2228591 | MAF | 0.035/0.019 | 0.140/0.142 | 0.062/0.067 | 0.052/0.052 | 0.067/0.061 |  |  |  |
|  |  | OR(95%CI) | 1.97(1.04-3.74) | 1.02(0.64-1.63) | 0.92(0.63-1.36) | 0.97(0.60-1.57) | 1.09(0.75-1.58) | 1.06(0.87-1.29) | 0.55 | 0.41 |
| **NCOA3** | Arg218Cys | HWE | 0.99/0.99 | 0.99/0.99 | -/- | 0.23/0.47 | <0.01/0.26 |  |  |  |
|  | rs6094752 | MAF | 0.072/0.079 | 0.019/0.021 | 0/0 | 0.028/0.040 | 0.045/0.048 |  |  |  |
|  |  | OR(95%CI) | 0.90(0.61-1.33) | 0.85(0.27-2.72) | - | 0.70(0.40-1.25) | 0.95(0.61-1.48) | 0.88(0.68-1.13) | 0.30 | 0.85 |
|  | Met391Val | HWE | 0.99/0.99 | -/- | -/- | -/- | -/- |  |  |  |
|  |  | MAF | 0.015/0.016 | 0/0 | 0/0 | 0/0 | 0/0 |  |  |  |
|  |  | OR(95%CI) | 0.88(0.38-2.01) | - | - | - | - | 0.93(0.41-2.13) | 0.86 |  |
|  | Pro559Ser | HWE | 0.99/0.99 | -/0.99 | -/- | 0.99/- | 0.99/- |  |  |  |
|  | rs2230781 | MAF | 0.038/0.037 | 0/0.003 | 0/0 | 0.003/0 | 0.003/0 |  |  |  |
|  |  | OR(95%CI) | 1.04(0.60-1.79) | - | - | - | - | 1.13(0.68-1.89) | 0.64 | 0.99 |
|  | Gln586His | HWE | 0.99/0.99 | 0.05/0.99 | 0.50/0.99 | 0.49/0.38 | 0.99/0.11 |  |  |  |
|  | rs2230782 | MAF | 0.023/0.020 | 0.024/0.049 | 0.041/0.045 | 0.086/0.063 | 0.075/0.086 |  |  |  |
|  |  | OR(95%CI) | 1.16(0.58-2.34) | 0.45(0.17-1.19) | 0.89(0.55-1.44) | 1.44(0.96-2.18) | 0.87(0.61-1.24) | 1.00(0.80-1.23) | 0.97 | 0.15 |
|  | Ser662Phe | HWE | 0.99/- | -/- | 0.99/- | -/- | -/- | -/- |  |  |
|  |  | MAF | 0.001/0 | 0/0 | 0.001/0 | 0/0 | 0/0 |  |  |  |
|  |  | OR(95%CI) | - | - | - | - | - | - |  |  |
| **FOXA1** | Ala83Thr | HWE | 0.38/0.16 | 0.12/0.14 | 0.99/0.34 | 0.81/0.91 | 0.76/0.61 |  |  |  |
|  | rs7144658 | MAF | 0.521/0.580 | 0.159/0.188 | 0.127/0.118 | 0.349/0.333 | 0.430/0.392 |  |  |  |
|  |  | OR(95%CI) | 0.79(0.64-0.97) | 0.82(0.54-1.23) | 1.08(0.81-1.45) | 1.07(0.86-1.34) | 1.14(0.93-1.39) | 0.99(0.89-1.10) | 0.85 | 0.07 |
|  | Ser448Asn | HWE | 0.99/0.23 | 0.99/0.99 | -/0.99 | <0.01/0.18 | 0.72/0.04 |  |  |  |
|  | rs33984772 | MAF | 0.014/0.025 | 0.023/0.023 | 0/0.002 | 0.050/0.046 | 0.077/0.057 |  |  |  |
|  |  | OR(95%CI) | 0.54(0.25-1.18) | 0.99(0.34-2.85) | - | 1.09(0.68-1.73) | 1.4(0.95-2.06) | 1.09(0.84-1.42) | 0.50 | 0.38 |
| **MPG** | Val242Leu | HWE | 0.99/0.99 | -/- | 0.99/- | -/0.99 | 0.99/- |  |  |  |
|  |  | MAF | 0.003/0.002 | 0/0 | 0.001/0 | 0/0.001 | 0.001/0 |  |  |  |
|  |  | OR(95%CI) | 1.29(0.18-9.27) | - | - | - | - | 1.62(0.36-7.29) | 0.53 |  |
| **NCOR1** | Val1996/1997del | HWE | 0.99/0.28 | -/- | -/- | 0.99/- | -/- |  |  |  |
|  |  | MAF | 0.024/0.028 | 0/0 | 0/0 | 0.003/0 | 0/0 |  |  |  |
|  |  | OR(95%CI) | 0.85(0.45-1.61) | - | - | - | - | 0.96(0.52-1.77) | 0.89 | 0.97 |
| **NCOR2** | Thr35Met | HWE | -/- | 0.99/0.99 | -/- | -/- | -/0.99 |  |  |  |
|  |  | MAF | 0/0 | 0.005/0.025 | 0/0 | 0/0 | 0/0.001 |  |  |  |
|  |  | OR(95%CI) | - | 0.20(0.03-1.56) | - | - | - | 0.18(0.02-1.37) | 0.10 | 0.97 |
|  | His52Arg | HWE | 0.01/0.23 | <0.01/- | -/- | -/0.99 | 0.99/- |  |  |  |
|  |  | MAF | 0.042/0.026 | 0.010/0 | 0/0 | 0/0.001 | 0.001/0 |  |  |  |
|  |  | OR(95%CI) | 1.59(0.91-2.78) | - | - | - | - | 1.79(1.05-3.05) | 0.03 |  |
|  | Gly781Glu | HWE | 0.01/0.45 | 0.04/0.99 | -/- | 0.08/0.48 | 0.27/0.82 |  |  |  |
|  | rs7978237 | MAF | 0.094/0.071 | 0.062/0.037 | 0/0.001 | 0.131/0.125 | 0.131/0.122 |  |  |  |
|  |  | OR(95%CI) | 1.31(0.91-1.88) | 1.70(0.85-3.39) | - | 1.05(0.77-1.44) | 1.15(0.85-1.55) | 1.16(0.97-1.39) | 0.10 | 0.74 |
|  | Lys980Thr | HWE | -/- | -/0.99 | 0.99/0.99 | -/- | -/- |  |  |  |
|  |  | MAF | 0/0 | 0/0.014 | 0.022/0.011 | 0/0 | 0/0 |  |  |  |
|  |  | OR(95%CI) | - | - | 2.03(0.90-4.57) | - | - | 1.33(0.67-2.67) | 0.42 | 0.96 |
|  | Ala995Gly | HWE | 0.99/0.73 | -/- | -/- | 0.99/0.99 | -/0.99 |  |  |  |
|  | rs11057592 | MAF | 0.075/0.078 | 0/0 | 0/0 | 0.009/0.008 | 0/0.003 |  |  |  |
|  |  | OR(95%CI) | 0.97(0.67-1.43) | - | - | 1.15(0.37-3.61) | - | 0.95(0.67-1.36) | 0.79 | 0.96 |
|  | Ser1525Thr | HWE | 0.17/0.29 | -/0.99 | -/- | 0.99/0.99 | -/- |  |  |  |
|  |  | MAF | 0.024/0.028 | 0/0.002 | 0/0 | 0.005/0.003 | 0/0 |  |  |  |
|  |  | OR(95%CI) | 0.87(0.47-1.61) | - | - | 1.67(0.28-10.13) | - | 0.91(0.51-1.62) | 0.75 | 0.77 |
|  | Ala1706Thr | HWE | 0.19/0.99 | <0.01/0.07 | 0.10/<0.01 | 0.02/0.16 | 0.31/0.72 |  |  |  |
|  | rs2229840 | MAF | 0.120/0.120 | 0.102/0.060 | 0.141/0.116 | 0.168/0.209 | 0.148/0.160 |  |  |  |
|  |  | OR(95%CI) | 1.01(0.74-1.38) | 1.56(0.92-2.62) | 1.23(0.94-1.63) | 0.78(0.60-1.01) | 0.87(0.67-1.15) | 0.99(0.86-1.13) | 0.83 | 0.06 |
|  | Ala2007Thr | HWE | 0.99/0.58 | 0.99/0.99 | 0.60/0.61 | 0.24/0.99 | 0.53/0.34 |  |  |  |
|  | rs2227277 | MAF | 0.036/0.045 | 0.014/0.012 | 0.047/0.049 | 0.030/0.035 | 0.044/0.031 |  |  |  |
|  |  | OR(95%CI) | 0.82(0.48-1.39) | 1.14(0.29-4.53) | 0.94(0.59-1.49) | 0.84(0.46-1.51) | 1.51(0.89-2.54) | 1.00(0.78-1.29) | 0.98 | 0.58 |
|  | Ala2011Val | HWE | 0.05/0.99 | -/0.99 | -/- | -/- | -/- |  |  |  |
|  |  | MAF | 0.014/0.011 | 0/0.002 | 0/0 | 0/0 | 0/0 |  |  |  |
|  |  | OR(95%CI) | 1.30(0.53-3.16) | - | - | - | - | 1.15(0.48-2.74) | 0.76 |  |
|  | Thr2216Pro | HWE | 0.22/0.99 | -/0.99 | -/- | -/0.99 | -/0.99 |  |  |  |
|  | rs1472840 | MAF | 0.028/0.022 | 0/0.005 | 0/0 | 0/0.001 | 0/0.001 |  |  |  |
|  |  | OR(95%CI) | 1.29(0.67-2.48) | - | - | - | - | 1.04(0.56-1.93) | 0.90 |  |
|  | Ser2311Gly | HWE | 0.99/0.99 | 0.35/0.25 | 0.99/0.12 | 0.99/0.99 | 0.99/0.99 |  |  |  |
|  | rs2228587 | MAF | 0.006/0.009 | 0.068/0.060 | 0.029/0.039 | 0.005/0.001 | 0.006/0.005 |  |  |  |
|  |  | OR(95%CI) | 0.63(0.19-2.13) | 1.11(0.58-2.11) | 0.76(0.45-1.30) | 3.53(0.37-34.16) | 1.26(0.33-4.84) | 0.93(0.64-1.34) | 0.68 | 0.59 |
|  | Ala2496Thr | HWE | 0.99/0.08 | 0.47/0.28 | 0.99/0.45 | <0.01/- | 0.99/0.99 |  |  |  |
|  |  | MAF | 0.008/0.014 | 0.079/0.062 | 0.027/0.038 | 0.005/0 | 0.008/0.003 |  |  |  |
|  |  | OR(95%CI) | 0.58(0.21-1.59) | 1.25(0.69-2.29) | 0.71(0.41-1.25) | - | 1.77(0.44-7.18) | 0.96(0.68-1.38) | 0.84 | 0.43 |
| **CALCOCO1** | Arg12His | HWE | 0.99/0.99 | 0.99/0.99 | -/- | 0.99/0.99 | 0.99/0.99 |  |  |  |
|  |  | MAF | 0.012/0.005 | 0.009/0.002 | 0/0 | 0.002/0.001 | 0.006/0.003 |  |  |  |
|  |  | OR(95%CI) | 2.76(0.81-9.34) | 6.02(0.52-69.11) | - | 1.10(0.07-17.77) | 1.77(0.41-7.68) | 2.29(1.00-5.26) | 0.05 | 0.86 |
|  | Arg393Lys | HWE | 0.09/0.69 | 0.99/0.06 | 0.10/0.99 | 0.83/0.60 | 0.42/0.23 |  |  |  |
|  | rs3741659 | MAF | 0.128/0.14 | 0.276/0.246 | 0.338/0.365 | 0.149/0.179 | 0.147/0.143 |  |  |  |
|  |  | OR(95%CI) | 0.91(0.67-1.21) | 1.19(0.81-1.77) | 0.89(0.73-1.09) | 0.80(0.60-1.07) | 0.99(0.74-1.32) | 0.93(0.82-1.05) | 0.21 | 0.50 |
|  | Ala527Thr | HWE | 0.99/0.99 | -/- | -/- | -/0.99 | -/- |  |  |  |
|  |  | MAF | 0.015/0.014 | 0/0 | 0/0 | 0/0.003 | 0/0 |  |  |  |
|  |  | OR(95%CI) | 1.09(0.46-2.57) | - | - | - | - | 0.92(0.41-2.11) | 0.85 |  |
|  | Gly561Val | HWE | 0.99/0.99 | -/- | -/- | 0.99/0.99 | 0.99/0.99 |  |  |  |
|  | rs34229062 | MAF | 0.007/0.005 | 0/0.003 | 0/0 | 0.008/0.001 | 0.006/0.011 |  |  |  |
|  |  | OR(95%CI) | 1.66(0.44-6.28) | - | - | 6.05(0.70-52.15) | 0.61(0.20-1.87) | 1.17(0.57-2.39) | 0.67 | 0.29 |
|  | Thr639Pro | HWE | 0.38/0.99 | -/0.99 | -/- | 0.99/0.99 | -/- |  |  |  |
|  | rs34281379 | MAF | 0.065/0.067 | 0/0.01 | 0/0 | 0.006/0.003 | 0/0 |  |  |  |
|  |  | OR(95%CI) | 0.97(0.64-1.48) | - | - | 2.15(0.39-11.92) | - | 0.95(0.64-1.42) | 0.82 | 0.71 |
| **CREBBP** | Pro858Ser | HWE | 0.99/0.99 | -/- | -/- | 0.99/- | -/- |  |  |  |
|  |  | MAF | 0.003/0.006 | 0/0 | 0/0 | 0.002/0 | 0/0 |  |  |  |
|  |  | OR(95%CI) | 0.49(0.09-2.56) | - | - | - | - | 0.67(0.16-2.81) | 0.58 |  |
|  | Thr910Ala | HWE | -/0.99 | -/- | -/0.99 | -/- | 0.99/0.99 |  |  |  |
|  |  | MAF | 0/0.001 | 0/0 | 0/0.001 | 0/0 | 0.005/0.002 |  |  |  |
|  |  | OR(95%CI) | - | - | - | - | 1.93(0.34-10.89) | 1.11(0.27-4.48) | 0.89 |  |
|  | Val992Ile | HWE | 0.99/0.99 | -/- | -/- | 0.99/- | 0.99/0.99 |  |  |  |
|  |  | MAF | 0.038/0.036 | 0/0 | 0/0 | 0.003/0 | 0.001/0.001 |  |  |  |
|  |  | OR(95%CI) | 1.06(0.62-1.83) | - | - | - | 1.01(0.06-16.36) | 1.14(0.68-1.92) | 0.62 | 0.99 |
|  | Gly2229Ser | HWE | 0.99/0.99 | -/- | -/- | -/- | 0.99/- |  |  |  |
|  |  | MAF | 0.015/0.011 | 0/0 | 0/0 | 0/0 | 0.003/0 |  |  |  |
|  |  | OR(95%CI) | 1.41(0.57-3.53) | - | - | - | - | 1.69(0.70-4.07) | 0.24 |  |
| **SMARCA2** | Asp1546Glu | HWE | 0.17/0.90 | 0.73/0.50 | 0.41/<0.01 | 0.99/0.43 | 0.99/0.83 |  |  |  |
|  | rs2296212 | MAF | 0.273/0.242 | 0.163/0.160 | 0.179/0.180 | 0.209/0.201 | 0.131/0.129 |  |  |  |
|  |  | OR(95%CI) | 1.18(0.94-1.48) | 1.06(0.68-1.65) | 0.99(0.77-1.28) | 1.05(0.82-1.36) | 1.01(0.75-1.36) | 1.07(0.94-1.21) | 0.29 | 0.87 |

^a^SNPs without an RS# have not been reported in dbSNP.

^b^ORs for gene dosage effects adjusted for age and race(pooled ORs).

^c^P-value for allele dosage effects (i.e. 0,1,2 copies of the variant allele, 1 df test).

^d^P-value for racial/ethnic heterogeneity of effects (4 df test).

**Supplementary Table 2. Descriptive Characteristics of Breast Cancer Cases and Controls in the MEC.**

|  |  |  | **Cases/Controls** |  |  |
| --- | --- | --- | --- | --- | --- |
|  | **African**  **Americans** | **Native**  **Hawaiians** | **Japanese**  **Americans** | **Latinos** | **European**  **Americans** |
| **n** | **345/426** | **108/290** | **425/419** | **334/386** | **400/440** |
| **Age (median, yrs)** | 66/65 | 60/58 | 66/65 | 64/64 | 66/61 |
| **Menopausal Status (%)** |  |  |  |  |  |
| **Premenopausal** | 13/10 | 19/26 | 12/19 | 9/9 | 7/20 |
| **Postmenopausal^a^** | 79/84 | 73/70 | 84/77 | 83/85 | 90/79 |
| **Unknown** | 8/6 | 8/4 | 4/4 | 8/6 | 3/1 |
| **Body Mass Index (%)^a,b^** |  |  |  |  |  |
| **<25** | 29/21 | 34/37 | 63/69 | 30/32 | 56/51 |
| **≥25** | 69/77 | 66/61 | 35/31 | 70/66 | 44/49 |
| **Ever use of hormone therapy (%)^a,b^** | |  |  |  |  |
| **Never** | 47/49 | 38/38 | 25/28 | 41/42 | 23/32 |
| **Past** | 27/23 | 16/23 | 14/15 | 19/21 | 16/16 |
| **Current** | 22/25 | 42/39 | 60/53 | 35/31 | 58/51 |
| **Age at menarche (yrs, %)** |  |  |  |  |  |
| **<12** | 54/44 | 52/59 | 56/51 | 47/46 | 55/49 |
| **13-14** | 35/42 | 31/29 | 32/34 | 38/39 | 36/43 |
| **14+** | 10/13 | 14/11 | 14/11 | 12/13 | 9/8 |
| **First degree family history of breast cancer** | | |  |  |  |
| **Yes (%)** | 20/13 | 19/14 | 17/11 | 15/10 | 16/9 |
| **Advanced breast cancer (%)^c^** | 29 | 27 | 21 | 30 | 26 |
| **Estrogen receptor status (%)** |  |  |  |  |  |
| **Positive** | 56 | 79 | 76 | 60 | 73 |
| **Negative** | 26 | 14 | 13 | 22 | 16 |
| **Unknown** | 18 | 7 | 11 | 19 | 12 |
|  |  |  |  |  |  |

^a^Postmenopausal is defined as a woman who reported natural menopause or a bilateral oophorectomy.

^b^Among postmenopausal women.

^c^Advanced disease defined as non-localized, SEER stage 2-7.

Note: not all values add up to 100% due to missing data.

**Supplementary Table 3. Genotype Counts for Breast Cancer Cases and Controls.**

| **Gene/**  **Variant** | **Genotype Counts (AA, Aa, aa)^a^** | | | | | |
| --- | --- | --- | --- | --- | --- | --- |
|  | **Controls** | **Cases** | **ER+ Cases** | **ER- Cases** | **Localized Cases** | **Regional/Metastatic Cases** |
| **EP300** |  |  |  |  |  |  |
| Ser507Gly | 1928/23/0 | 1587/18/1 | 1075/11/0 | 285/6/1 | 1167/13/1 | 415/5/0 |
| Ile997Val | 1268/530/154 | 1065/417/118 | 743/272/69 | 179/83/28 | 788/306/86 | 274/109/32 |
| Pro1986Leu | 1947/6/0 | 1603/3/0 | 1083/3/0 | 291/0/0 | 1179/2/0 | 419/1/0 |
| Gln2223Pro | 1882/56/1 | 1540/48/1 | 1046/31/1 | 278/8/0 | 1136/31/1 | 399/17/0 |
| **CCND1** |  |  |  |  |  |  |
| Pro241Pro | 658/889/343 | 516/740/291 | 323/512/214 | 108/117/52 | 374/552/215 | 140/186/75 |
| **NCOA1** |  |  |  |  |  |  |
| Pro1272Ser | 1913/35/1 | 1573/31/1 | 1065/21/0 | 286/6/0 | 1155/26/1 | 413/5/0 |
| **NCOA2** |  |  |  |  |  |  |
| Ala407Ser | 1947/5/0 | 1595/8/0 | 1082/4/0 | 286/3/0 | 1178/3/0 | 412/5/0 |
| Asn1212Ser | 1934/11/0 | 1598/10/0 | 1083/7/0 | 288/3/0 | 1177/6/0 | 416/4/0 |
| Met1282Ile | 1712/216/15 | 1406/181/6 | 944/125/6 | 261/30/0 | 1030/139/5 | 372/41/1 |
| **NCOA3** |  |  |  |  |  |  |
| Arg218Cys | 1799/142/5 | 1501/96/6 | 1019/62/4 | 269/20/1 | 1114/63/3 | 382/33/3 |
| Met391Val | 1941/14/0 | 1600/10/0 | 1082/7/0 | 291/1/0 | 1178/7/0 | 417/3/0 |
| Pro559Ser | 1919/33/0 | 1579/30/0 | 1066/22/0 | 287/5/0 | 1160/25/0 | 414/5/0 |
| Gln586His | 1733/191/6 | 1432/162/5 | 968/110/3 | 258/33/1 | 1047/124/5 | 380/38/0 |
| Ser662Phe | 1950/0/0 | 1608/2/0 | 1088/2/0 | 292/0/0 | 1184/1/0 | 419/1/0 |
| **FOXA1** |  |  |  |  |  |  |
| Ala83Thr | 908/753/259 | 734/609/216 | 513/414/134 | 120/104/55 | 558/454/138 | 174/155/75 |
| Ser448Asn | 1795/105/7 | 1462/92/7 | 981/73/5 | 273/11/1 | 1090/59/5 | 368/33/2 |
| **MPG** |  |  |  |  |  |  |
| Val242Leu | 1937/3/0 | 1583/4/0 | 1073/4/0 | 286/0/0 | 1166/1/0 | 412/3/0 |
| **NCOR1** |  |  |  |  |  |  |
| Val1996/1997del | 1907/22/1 | 1546/18/0 | 1042/10/0 | 281/6/0 | 1137/11/0 | 404/7/0 |
| **NCOR2** |  |  |  |  |  |  |
| Thr35Met | 1921/15/0 | 1520/1/0 | 1030/1/0 | 275/0/0 | 1117/0/0 | 398/1/0 |
| His52Arg | 1881/19/1 | 1522/21/4 | 1042/8/3 | 269/7/1 | 1122/15/1 | 395/6/3 |
| Gly783Glu | 1638/254/12 | 1318/205/27 | 903/131/20 | 239/36/5 | 976/147/22 | 339/56/5 |
| Lys980Thr | 1922/17/0 | 1511/18/0 | 1021/12/0 | 270/5/0 | 1110/15/0 | 396/3/0 |
| Ala995Gly | 1782/69/3 | 1532/53/2 | 1042/33/0 | 271/13/2 | 1132/39/1 | 396/13/1 |
| Ser1525Thr | 1910/25/1 | 1559/17/1 | 1058/9/0 | 278/6/1 | 1150/10/1 | 404/7/0 |
| Ala1706Thr | 1449/417/54 | 1149/332/50 | 782/216/37 | 207/65/8 | 852/242/35 | 295/88/15 |
| Ala2007Thr | 1797/135/2 | 1437/112/3 | 982/71/1 | 260/21/0 | 1059/83/3 | 373/29/0 |
| Ala2011Val | 1901/10/0 | 1537/7/1 | 1036/6/1 | 278/1/0 | 1132/5/0 | 400/2/1 |
| Thr2216Pro | 1882/23/0 | 1538/16/1 | 1045/10/1 | 276/4/0 | 1131/12/1 | 402/4/0 |
| Ser2311Gly | 1842/70/4 | 1516/47/1 | 1020/33/1 | 279/6/0 | 1107/41/1 | 404/6/0 |
| Ala2496Thr | 1844/73/4 | 1504/48/2 | 1012/36/2 | 279/5/0 | 1100/42/2 | 399/6/0 |
| **CALCOCO1** |  |  |  |  |  |  |
| Arg12His | 1936/9/0 | 1576/16/0 | 1065/13/0 | 289/1/0 | 1163/10/0 | 408/6/0 |
| Arg393Lys | 1184/629/89 | 1007/461/83 | 669/324/57 | 187/77/18 | 747/341/62 | 257/118/21 |
| Ala527Thr | 1928/14/0 | 1591/10/0 | 1079/5/0 | 290/1/0 | 1170/9/0 | 416/1/0 |
| Gly561Val | 1927/16/0 | 1574/15/0 | 1067/11/0 | 286/2/0 | 1158/11/0 | 411/4/0 |
| Thr639Pro | 1871/62/1 | 1550/48/0 | 1052/29/0 | 282/8/0 | 1145/30/0 | 400/18/0 |
| **CREBBP** |  |  |  |  |  |  |
| Pro858Ser | 1900/5/0 | 1586/3/0 | 1075/2/0 | 287/1/0 | 1167/2/0 | 414/1/0 |
| Thr910Ala | 1946/4/0 | 1598/4/0 | 1080/4/0 | 290/0/0 | 1178/3/0 | 415/1/0 |
| Val992Ile | 1920/32/0 | 1572/29/0 | 1075/9/0 | 277/13/0 | 1158/21/0 | 409/8/0 |
| Gly2229Ser | 1928/9/0 | 1585/12/0 | 1074/7/0 | 288/2/0 | 1168/8/0 | 412/4/0 |
| **SMARCA2** |  |  |  |  |  |  |
| Asp1546Glu | 1282/593/58 | 1045/471/69 | 697/335/44 | 197/73/15 | 769/349/48 | 273/120/21 |

^a^AA=Homozygous wildtype; Aa=Heterozygotes; aa=Homozygous variant

**Supplementary Table 4. Effect Modification by Age at Menarche.**

|  | **Age at Menarche** | |  | |
| --- | --- | --- | --- | --- |
| **Gene/**  **Variant** | **≤12**  **cases/controls**  **854/966** | **>12**  **cases/controls**  **728/977** | | **P_Int_** |
| **EP300** |  |  | |  |
| Ser507Gly | 1.34(0.58-3.09) | 0.75(0.30-1.87) | | 0.41 |
| Ile997Val | 0.94(0.79-1.13) | 0.91(0.76-1.09) | | 0.77 |
| Pro1986Leu | 0.90(0.21-3.99) | - | |  |
| Gln2223Pro | 1.12(0.66-1.90) | 0.98(0.55-1.74) | | 0.79 |
| **CCND1** |  |  | |  |
| Pro241Pro | 1.07(0.93-1.23) | 1.08(0.94-1.25) | | 0.51 |
| **NCOA1** |  |  | |  |
| Pro1272Ser | 0.99(0.49-1.98) | 1.10(0.57-2.11) | | 0.77 |
| **NCOA2** |  |  | |  |
| Ala407Ser | 3.64(0.72-18.34) | 1.11(0.18-6.73) | | 0.31 |
| Asn1212Ser | 0.98(0.28-3.44) | 1.18(0.36-3.94) | | 0.99 |
| Met1282Ile | 1.05(0.79-1.40) | 1.09(0.82-1.43) | | 0.57 |
| **NCOA3** |  |  | |  |
| Arg218Cys | 1.00(0.70-1.45) | 0.75(0.53-1.08) | | 0.20 |
| Met391Val | 0.41(0.10-1.62) | 1.92(0.63-5.86) | | 0.15 |
| Pro559Ser | 0.81(0.40-1.65) | 1.65(0.77-3.52) | | 0.38 |
| Gln586His | 0.91(0.67-1.22) | 1.11(0.82-1.51) | | 0.27 |
| Ser662Phe | - | - | |  |
| **FOXA1** |  |  | |  |
| Ala83Thr | 0.97(0.83-1.12) | 1.02(0.87-1.19) | | 0.81 |
| Ser448Asn | 0.87(0.60-1.25) | 1.36(0.94-1.98) | | 0.07 |
| **MPG** |  |  | |  |
| Val242Leu | - | - | |  |
| **NCOR1** |  |  | |  |
| Val1996/1997del | 1.62(0.57-4.65) | 0.90(0.40-2.04) | | 0.25 |
| **NCOR2** |  |  | |  |
| Thr35Met | 0.40(0.05-3.33) | - | |  |
| His52Arg | 1.59(0.77-3.30) | 2.03(0.92-4.47) | | 0.85 |
| Gly783Glu | 1.11(0.87-1.42) | 1.21(0.93-1.58) | | 0.55 |
| Lys980Thr | 1.25(0.44-3.53) | 1.38(0.53-3.59) | | 0.84 |
| Ala995Gly | 1.06(0.65-1.72) | 0.79(0.46-1.36) | | 0.21 |
| Ser1525Thr | 0.69(0.33-1.45) | 1.21(0.47-3.12) | | 0.53 |
| Ala1706Thr | 1.05(0.87-1.27) | 0.92(0.76-1.12) | | 0.35 |
| Ala2007Thr | 1.27(0.88-1.82) | 0.76(0.52-1.10) | | 0.041 |
| Ala2011Val | 1.31(0.39-4.47) | 0.94(0.26-3.43) | | 0.56 |
| Thr2216Pro | 0.75(0.32-1.74) | 1.42(0.56-3.56) | | 0.48 |
| Ser2311Gly | 1.16(0.68-1.99) | 0.70(0.41-1.20) | | 0.26 |
| Ala2496Thr | 1.07(0.61-1.89) | 0.86(0.53-1.37) | | 0.75 |
| **CALCOCO1** |  |  | |  |
| Arg12His | 1.94(0.61-6.14) | 2.87(0.85-9.67) | | 0.67 |
| Arg393Lys | 0.92(0.77-1.09) | 0.96(0.80-1.14) | | 0.63 |
| Ala527Thr | 0.74(0.28-2.00) | 1.16(0.26-5.24) | | 0.79 |
| Gly561Val | 0.74(0.31-1.74) | 3.12(0.76-12.78) | | 0.094 |
| Thr639Pro | 1.17(0.63-2.18) | 0.82(0.48-1.40) | | 0.20 |
| **CREBBP** |  |  | |  |
| Pro858Ser | 0.86(0.05-13.94) | 0.68(0.12-3.75) | | 0.79 |
| Thr910Ala | 0.97(0.19-4.86) | 1.39(0.08-22.91) | | 0.85 |
| Val992Ile | 0.90(0.40-2.02) | 1.59(0.79-3.21) | | 0.55 |
| Gly2229Ser | 1.87(0.61-5.70) | 1.11(0.25-5.04) | | 0.47 |
| **SMARCA2** |  |  | |  |
| Asp1546Glu | 1.02(0.86-1.22) | 1.10(0.93-1.32) | | 0.64 |

ORs for gene dosage effects adjusted for age and race.

**Supplementary Table 5. Effect Modification by Body Mass Index (BMI)**

**Among Postmenopausal Women.**

|  | **BMI (kg/m^2^)** | |  |
| --- | --- | --- | --- |
| **Gene/**  **Variant** | **<25**  **cases/controls**  **855/966** | **≥ 25**  **cases/controls**  **561/749** | **P_Int_** |
| **EP300** |  |  |  |
| Ser507Gly | 1.65(0.66-4.13) | 0.96(0.36-2.57) | 0.63 |
| Ile997Val | 0.81(0.65-1.01) | 1.00(0.84-1.20) | 0.21 |
| Pro1986Leu | 1.57(0.08-29.17) | 0.31(0.03-2.96) | 0.46 |
| Gln2223Pro | 1.09(0.58-2.04) | 0.87(0.50-1.53) | 0.45 |
| **CCND1** |  |  |  |
| Pro241Pro | 1.12(0.95-1.32) | 1.09(0.94-1.27) | 0.84 |
| **NCOA1** |  |  |  |
| Pro1272Ser | 1.20(0.53-2.72) | 0.98(0.52-1.87) | 0.57 |
| **NCOA2** |  |  |  |
| Ala407Ser | 1.18(0.07-19.44) | 1.20(0.26-5.46) | 0.87 |
| Asn1212Ser | 1.00(0.14-7.32) | 1.17(0.35-3.89) | 0.87 |
| Met1282Ile | 1.11(0.80-1.54) | 0.96(0.71-1.29) | 0.65 |
| **NCOA3** |  |  |  |
| Arg218Cys | 1.04(0.67-1.62) | 0.73(0.50-1.05) | 0.10 |
| Met391Val | 0.67(0.11-4.15) | 0.84(0.24-2.92) | 0.88 |
| Pro559Ser | 0.42(0.11-1.70) | 1.31(0.68-2.51) | 0.32 |
| Gln586His | 1.12(0.79-1.59) | 1.04(0.76-1.43) | 0.79 |
| Ser662Phe | - | - |  |
| **FOXA1** |  |  |  |
| Ala83Thr | 1.18(0.98-1.43) | 0.85(0.73-1.00) | 0.00054 |
| Ser448Asn | 1.33(0.85-2.09) | 1.06(0.72-1.56) | 0.41 |
| **MPG** |  |  |  |
| Val242Leu | - | 1.40(0.19-10.03) |  |
| **NCOR1** |  |  |  |
| Val1996/1997del | 0.78(0.20-3.04) | 1.21(0.55-2.65) | 0.93 |
| **NCOR2** |  |  |  |
| Thr35Met | 0.60(0.07-5.42) | - |  |
| His52Arg | 1.26(0.46-3.45) | 1.84(0.88-3.85) | 0.86 |
| Gly783Glu | 1.16(0.85-1.59) | 1.09(0.84-1.40) | 0.46 |
| Lys980Thr | 1.41(0.59-3.38) | 0.47(0.05-4.85) | 0.50 |
| Ala995Gly | 0.86(0.41-1.82) | 0.94(0.57-1.53) | 0.59 |
| Ser1525Thr | 1.63(0.45-5.93) | 0.87(0.41-1.83) | 0.26 |
| Ala1706Thr | 1.01(0.81-1.27) | 0.97(0.80-1.18) | 0.81 |
| Ala2007Thr | 0.86(0.57-1.31) | 1.30(0.89-1.88) | 0.15 |
| Ala2011Val | - | 1.14(0.44-2.95) |  |
| Thr2216Pro | - | 1.33(0.66-2.70) |  |
| Ser2311Gly | 0.72(0.41-1.24) | 1.19(0.62-2.27) | 0.15 |
| Ala2496Thr | 0.83(0.49-1.42) | 1.12(0.60-2.08) | 0.41 |
| **CALCOCO1** |  |  |  |
| Arg12His | 1.62(0.47-5.63) | 3.13(0.92-10.61) | 0.55 |
| Arg393Lys | 0.94(0.77-1.14) | 0.90(0.74-1.09) | 0.78 |
| Ala527Thr | 2.19(0.19-24.83) | 0.82(0.30-2.27) | 0.33 |
| Gly561Val | 1.03(0.35-3.00) | 1.01(0.34-2.95) | 0.82 |
| Thr639Pro | 0.80(0.35-1.80) | 1.25(0.74-2.10) | 0.80 |
| **CREBBP** |  |  |  |
| Pro858Ser | 0.31(0.03-3.05) | 1.27(0.18-9.07) | 0.45 |
| Thr910Ala | - | 0.49(0.04-5.45) | 0.96 |
| Val992Ile | 0.99(0.37-2.64) | 1.41(0.69-2.87) | 0.98 |
| Gly2229Ser | 0.97(0.19-4.92) | 1.78(0.47-6.74) | 0.81 |
| **SMARCA2** |  |  |  |
| Asp1546Glu | 0.86(0.70-1.06) | 1.13(0.94-1.36) | 0.09 |

ORs for gene dosage effects adjusted for age and race.

**Supplementary Table** 6. Effect Modification by Hormone Therapy Use(E or E+P) Among Postmenopausal Women.

|  | **Hormone Therapy Use** | | |  |
| --- | --- | --- | --- | --- |
| **Gene/**  **Variant** | **Never Use**  **cases/controls**  **855/966** | **Past Use**  **cases/controls**  **561/749** | **Current Use**  **cases/controls**  **167/228** | **P_Int_** |
| **EP300** |  |  |  |  |
| Ser507Gly | 2.32(0.53-10.23) | 4.12(0.38-44.50) | 0.97(0.41-2.31) | 0.44 |
| Ile997Val | 0.90(0.71-1.13) | 0.80(0.58-1.11) | 1.03(0.84-1.28) | 0.34 |
| Pro1986Leu | 3.85(0.32-46.76) | - | - | 0.97 |
| Gln2223Pro | 1.14(0.53-2.43) | 1.20(0.44-3.29) | 0.96(0.53-1.73) | 0.93 |
| **CCND1** |  |  |  |  |
| Pro241Pro | 1.02(0.83-1.26) | 0.92(0.70-1.20) | 1.21(1.00-1.46) | 0.11 |
| **NCOA1** |  |  |  |  |
| Pro1272Ser | 1.15(0.49-2.70) | 1.39(0.50-3.87) | 0.74(0.33-1.67) | 0.63 |
| **NCOA2** |  |  |  |  |
| Ala407Ser | 1.56(0.38-6.37) | - | - | 0.97 |
| Asn1212Ser | 0.78(0.18-3.34) | 1.02(0.06-16.71) | 0.96(0.16-5.98) | 0.97 |
| Met1282Ile | 0.95(0.66-1.37) | 0.91(0.54-1.53) | 1.11(0.80-1.56) | 0.65 |
| **NCOA3** |  |  |  |  |
| Arg218Cys | 0.77(0.49-1.19) | 0.83(0.48-1.42) | 0.90(0.55-1.45) | 0.90 |
| Met391Val | 0.55(0.10-2.90) | 1.70(0.27-10.51) | 0.78(0.07-8.82) | 0.60 |
| Pro559Ser | 1.84(0.78-4.34) | 0.90(0.32-2.55) | 0.51(0.13-1.98) | 0.17 |
| Gln586His | 1.17(0.77-1.79) | 1.21(0.67-2.15) | 0.88(0.63-1.23) | 0.60 |
| Ser662Phe | - | - | - | 0.99 |
| **FOXA1** |  |  |  |  |
| Ala83Thr | 0.95(0.78-1.16) | 0.99(0.75-1.30) | 0.98(0.82-1.18) | 0.86 |
| Ser448Asn | 0.80(0.46-1.40) | 1.72(0.91-3.22) | 1.26(0.82-1.94) | 0.18 |
| **MPG** |  |  |  |  |
| Val242Leu | - | - | - | 0.97 |
| **NCOR1** |  |  |  |  |
| Val1996/1997del | 0.59(0.19-1.80) | 1.04(0.30-3.55) | 2.33(0.54-10.09) | 0.45 |
| **NCOR2** |  |  |  |  |
| Thr35Met | 0.47(0.05-4.23) | - | - | 0.99 |
| His52Arg | 1.81(0.89-3.71) | 0.87(0.26-2.86) | 2.01(0.59-6.80) | 0.67 |
| Gly783Glu | 1.31(0.94-1.81) | 1.00(0.64-1.57) | 1.09(0.79-1.50) | 0.68 |
| Lys980Thr | 1.82(0.34-9.67) | 0.79(0.12-5.21) | 1.16(0.42-3.22) | 0.76 |
| Ala995Gly | 0.93(0.51-1.70) | 0.90(0.42-1.97) | 0.83(0.37-1.90) | 0.77 |
| Ser1525Thr | 0.46(0.14-1.45) | 1.33(0.36-4.87) | 1.66(0.60-4.63) | 0.28 |
| Ala1706Thr | 1.07(0.82-1.38) | 0.78(0.55-1.10) | 1.00(0.79-1.26) | 0.36 |
| Ala2007Thr | 0.84(0.52-1.34) | 2.29(1.15-4.56) | 1.07(0.70-1.63) | 0.060 |
| Ala2011Val | 0.86(0.20-3.69) | 1.60(0.24-10.66) | 1.62(0.22-11.91) | 0.86 |
| Thr2216Pro | 1.23(0.56-2.73) | 4.96(0.54-45.67) | 1.26(0.32-4.88) | 0.46 |
| Ser2311Gly | 0.64(0.29-1.41) | 0.98(0.40-2.39) | 0.97(0.52-1.83) | 0.73 |
| Ala2496Thr | 0.77(0.37-1.60) | 0.98(0.42-2.30) | 1.05(0.56-1.97) | 0.82 |
| **CALCOCO1** |  |  |  |  |
| Arg12His | 2.59(0.60-11.18) | 2.21(0.20-25.05) | 1.60(0.44-5.81) | 0.80 |
| Arg393Lys | 0.76(0.60-0.97) | 0.85(0.61-1.18) | 1.07(0.87-1.30) | 0.10 |
| Ala527Thr | 0.59(0.11-3.12) | 1.54(0.34-7.09) | 0.68(0.12-3.85) | 0.58 |
| Gly561Val | 2.70(0.66-10.95) | 1.52(0.29-8.05) | 0.46(0.14-1.53) | 0.19 |
| Thr639Pro | 1.06(0.58-1.94) | 1.49(0.53-4.20) | 0.93(0.40-2.16) | 0.49 |
| **CREBBP** |  |  |  |  |
| Pro858Ser | - | 0.98(0.06-16.03) | 1.25(0.08-20.70) | 0.99 |
| Thr910Ala | - | - | 0.83(0.12-5.98) | 0.99 |
| Val992Ile | 1.24(0.53-2.87) | 0.63(0.14-2.75) | 2.37(0.74-7.60) | 0.63 |
| Gly2229Ser | 1.14(0.30-4.33) | 4.46(0.49-40.67) | - | 0.52 |
| **SMARCA2** |  |  |  |  |
| Asp1546Glu | 0.99(0.79-1.24) | 0.95(0.68-1.31) | 1.10(0.89-1.37) | 0.80 |

ORs for gene dosage effects adjusted for age and race.
